# Supplementary material for: Intranasal Delivery of RGD-Containing Osteopontin Heptamer Peptide Confers Neuroprotection in the Ischemic Brain and Augments Microglia M2 Polarization
Source: Int J Mol Sci. 2021 Sep 16;22(18):9999. doi: 10.3390/ijms22189999 (PMC8466884; doi:10.3390/ijms22189999)
Supplement: Supplementary file 1 [file ijms-22-09999-s001.zip › raw data - Immunoblot.pptx]

## Slide 1
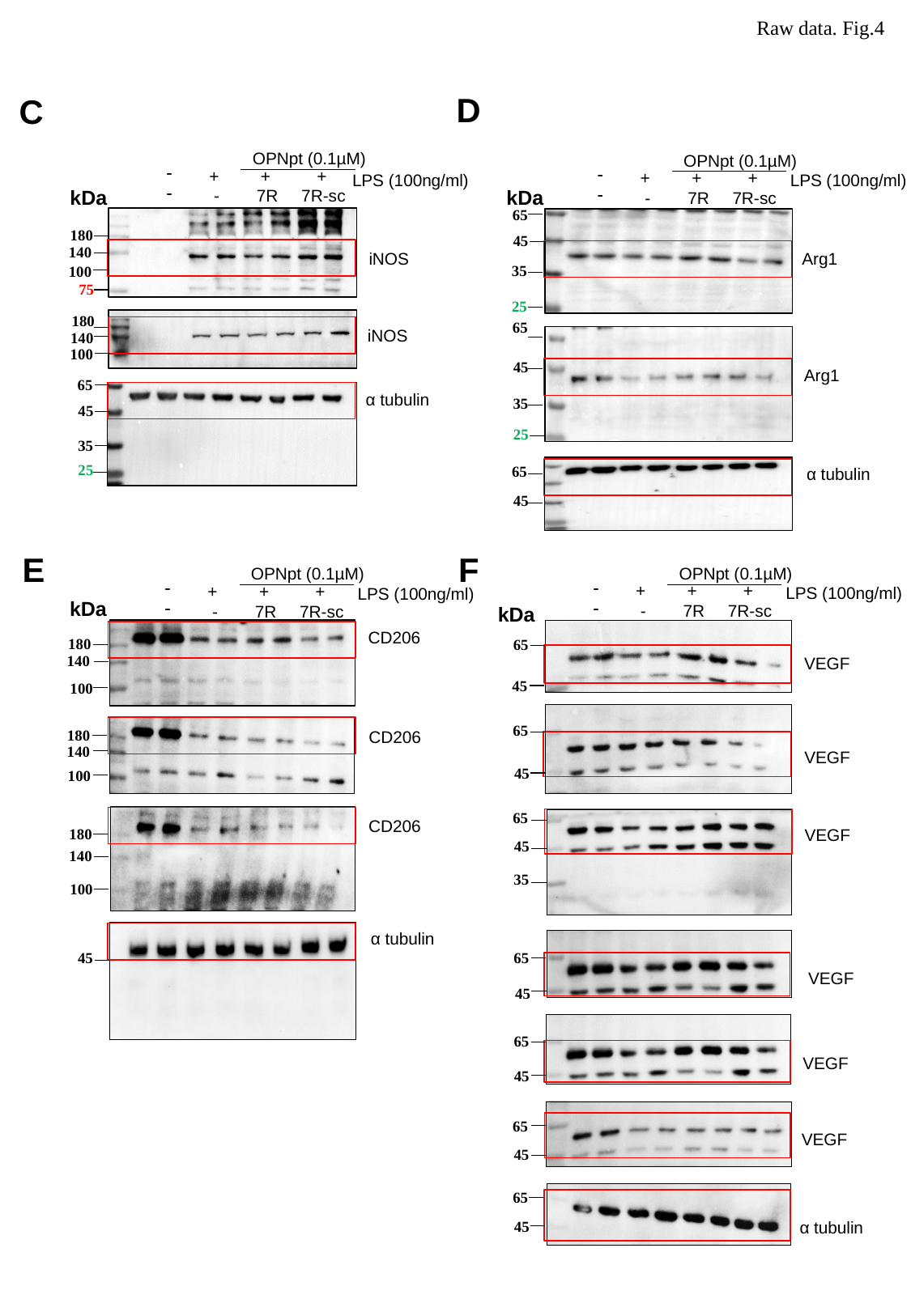

Raw data. Fig.4
D
C
OPNpt (0.1µM)
OPNpt (0.1µM)
 + + +
 - 7R 7R-sc
 + + +
 - 7R 7R-sc
LPS (100ng/ml)
LPS (100ng/ml)
kDa
kDa
65
180
45
140
Arg1
iNOS
35
100
75
25
180
65
iNOS
140
100
45
Arg1
65
α tubulin
35
45
25
35
25
65
α tubulin
45
F
E
OPNpt (0.1µM)
OPNpt (0.1µM)
 + + +
 - 7R 7R-sc
 + + +
 - 7R 7R-sc
LPS (100ng/ml)
LPS (100ng/ml)
kDa
kDa
CD206
180
65
140
VEGF
45
100
65
180
CD206
140
VEGF
45
100
65
CD206
180
VEGF
45
140
35
100
α tubulin
65
45
VEGF
45
65
VEGF
45
65
VEGF
45
65
45
α tubulin

## Slide 2
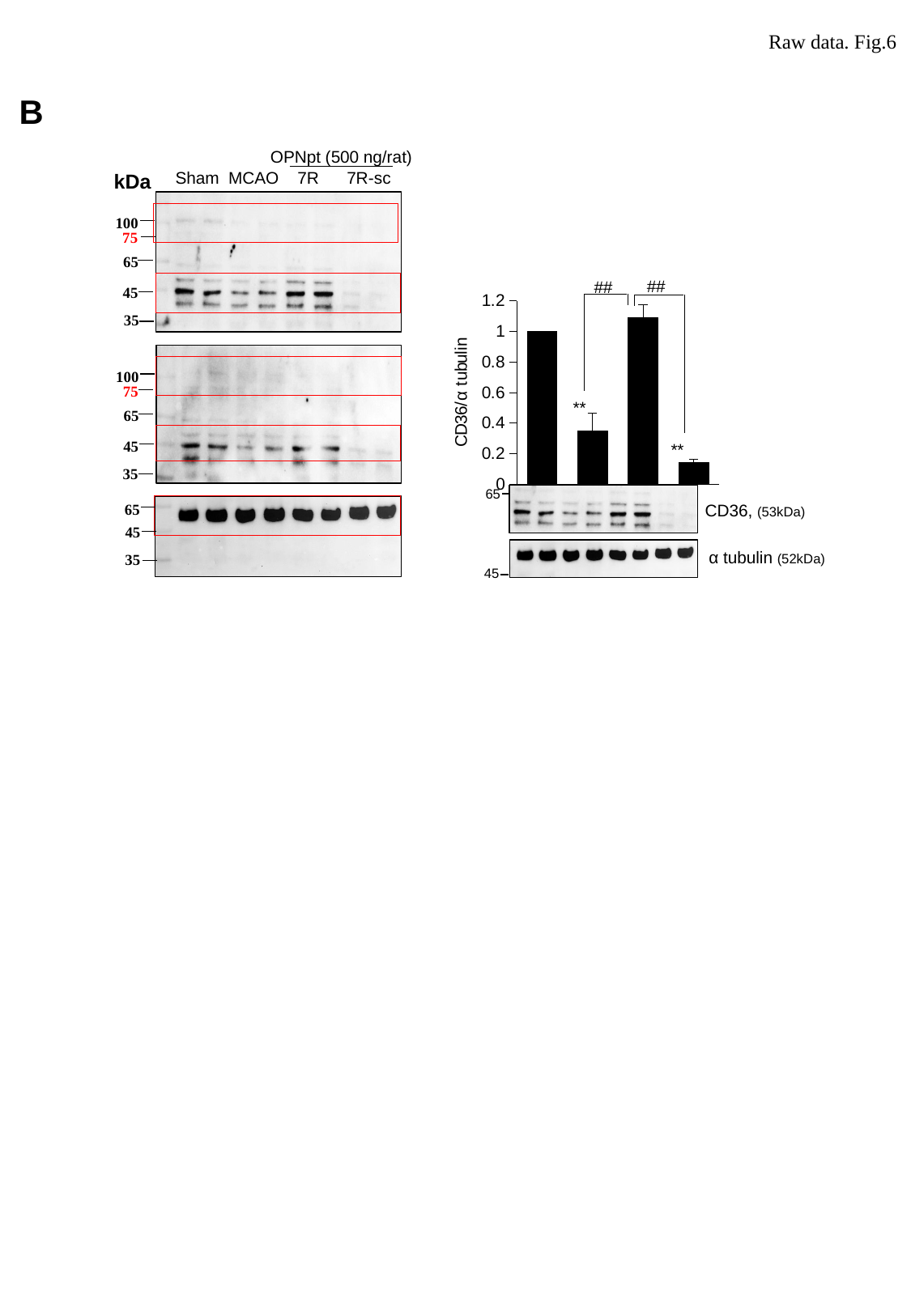

Raw data. Fig.6
B
OPNpt (500 ng/rat)
Sham MCAO 7R 7R-sc
kDa
100
75
65
##
##
45
### Chart
| Category | |
|---|---|35
100
75
**
65
45
**
35
65
65
CD36, (53kDa)
45
α tubulin (52kDa)
35
45
