# Supplementary material for: Intranasal Delivery of RGD-Containing Osteopontin Heptamer Peptide Confers Neuroprotection in the Ischemic Brain and Augments Microglia M2 Polarization
Source: Int J Mol Sci. 2021 Sep 16;22(18):9999. doi: 10.3390/ijms22189999 (PMC8466884; doi:10.3390/ijms22189999)
Supplement: Supplementary file 1 [file ijms-22-09999-s001.zip › Supplementary.pdf]

Supplementary Materials

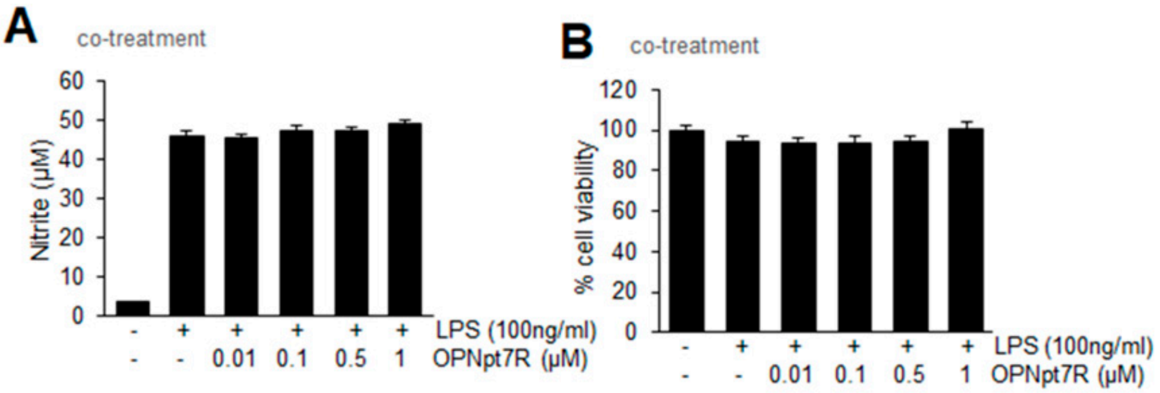

Figure S1. No anti-inflammatory effect of OPNpt7R co-treatment in primary microglial cultures. Primary microglial cultures were treated with OPNpt7R (0.01, 0.1, 0.5 or 1 μM) and LPS (100 ng/ml) for 24 h. Nitrite production was measured using the Griess assay **(A)** and cell viability was measured with MTT assay **(B)**. Results are presented as mean±SEM (*n* = 4).

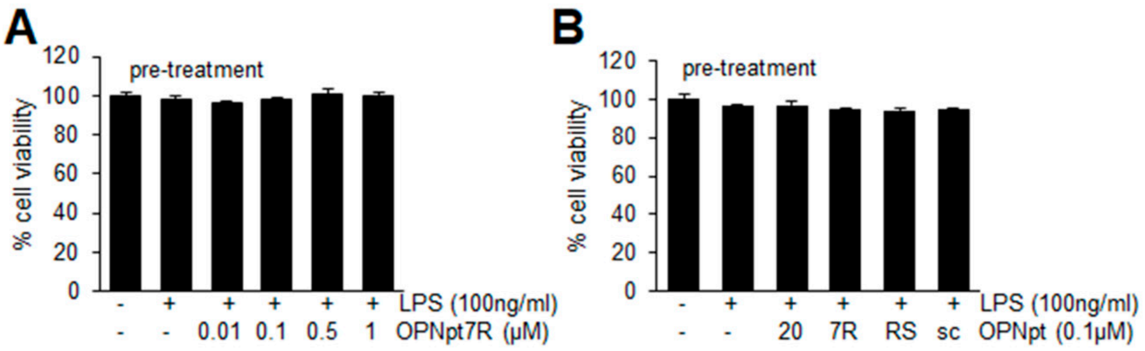

Figure S2. Cell viability of OPNpt7R-pre-treated primary microglial cultures after LPS treatment. Primary microglial cultures were pre-incubated with OPNpt7R (0.01, 0.1, 0.5 or 1 μM) for 1 h **(A)** or with 0.1 μM of OPNpt20, OPNpt7R, OPNpt7RS, or OPNpt7R-sc for 1 h **(B)** and treated with LPS (100 ng/ml) for 24 h. Cell viability was measured with MTT assay **(B)**. Results are presented as mean±SEM (*n* = 4).

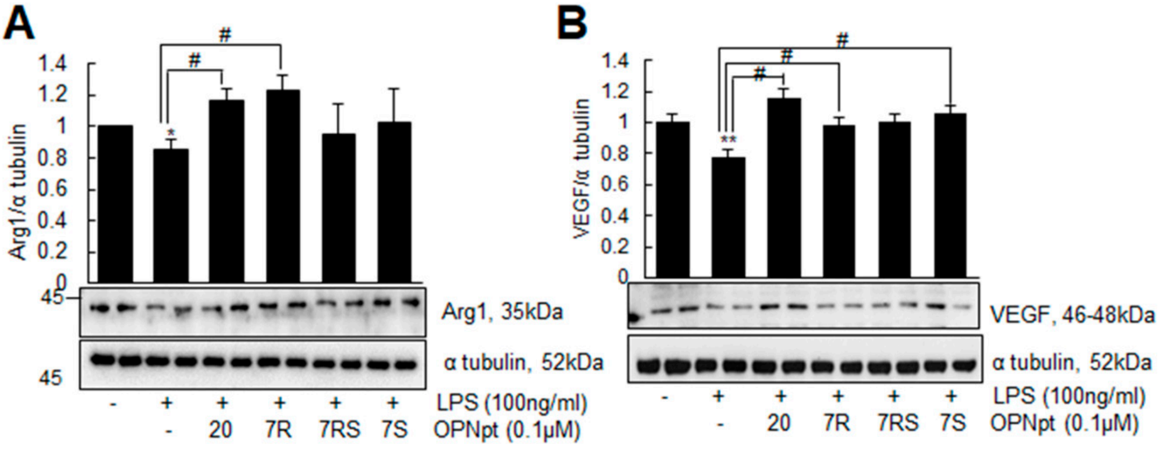

Figure S3. Comparison of anti-inflammatory effects of OPNpt7R with OPNpt20 and OPNpt7RS. Primary microglial cultures were pre-incubated with 0.1 μM of OPNpt20, OPNpt7R, or OPNpt7RS for 1 h and treated with LPS (100 ng/ml). Levels of Arg1 **(A)** and VEGF **(B)** were determined by

immunoblotting, and the results are presented as mean±SEM ($n = 3$). *$p < 0.05$, **$p < 0.01$ versus LPS-treated cells, #$p < 0.05$ between indicated groups.

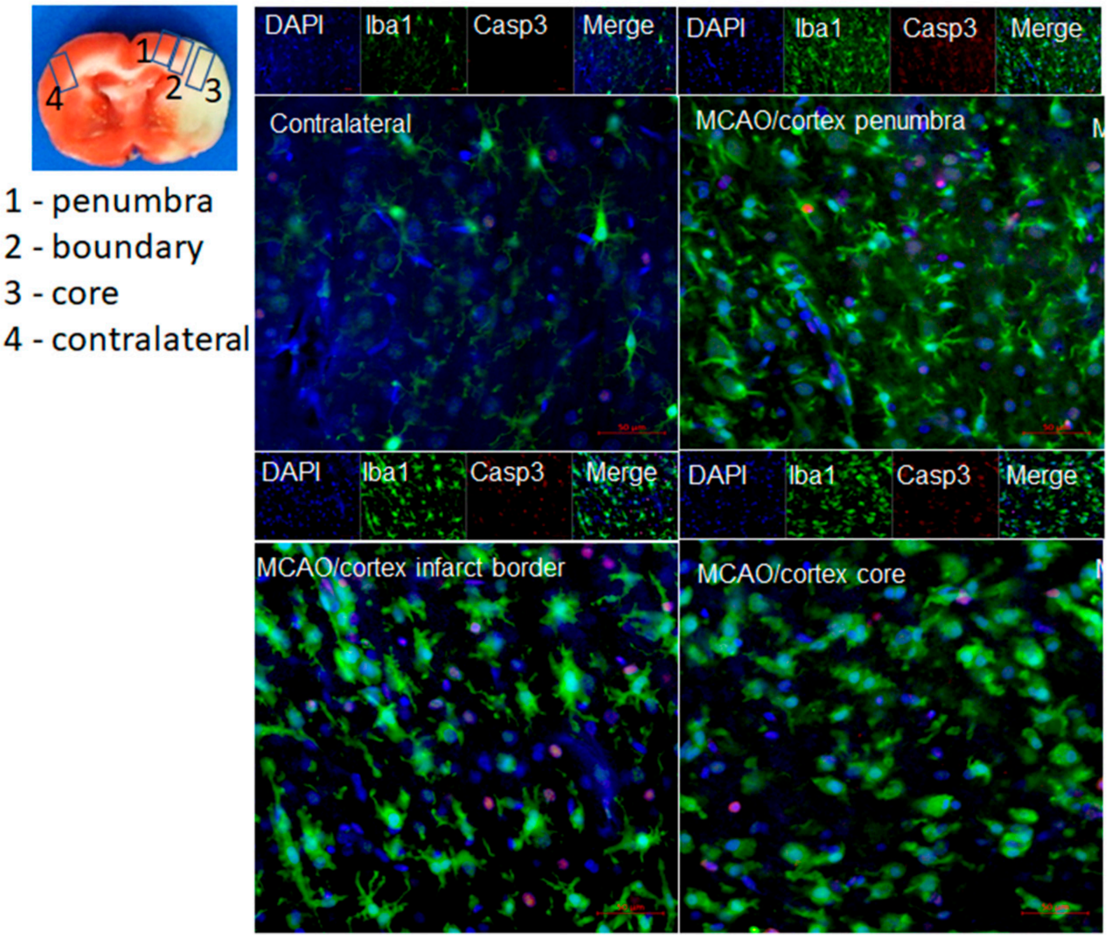

Figure S4. Activation of microglia in the cortical penumbra, infarct boundary, and infarct core of the post-ischemic brain. Coronal brain sections were prepared from 3 day after MCAO and processed for double immunofluorescence staining with anti-Iba1 and anti-activated caspase 3 antibodies and DAPI. Representative images were obtained from the cortical penumbra, infarct boundary, and cortical core of the ipsilateral hemisphere and also form contralateral hemisphere.

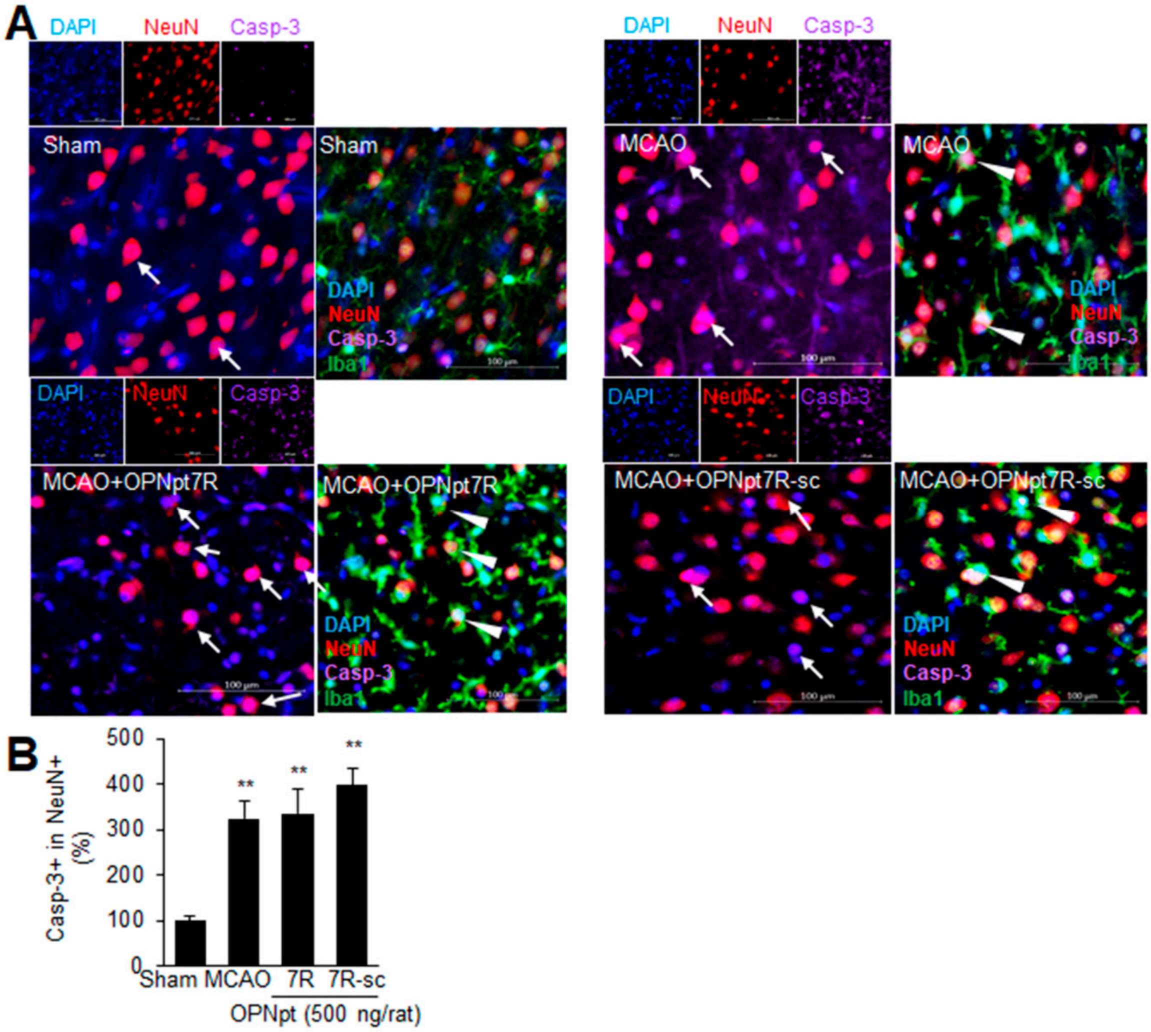

Figure S5. Activation of microglia in the cortical penumbra, infarct boundary, and infarct core of the post-ischemic brain. Coronal brain sections were prepared from 3 day after MCAO and processed for immunofluorescence staining with anti-neuN, anti-Iba1 and anti-activated caspase 3 antibodies and DAPI. Representative images were obtained from the cortical core of the ipsilateral hemisphere and the number of Caspase 3+ immunoreactivity in NeuN+ cells (arrows) were counted (0.2 mm^2) and presented as mean±SEM. Images in the right panel presented localization of Caspase 3+/NeuN+ immunoreactivity in Iba+ cells (arrow head).
